# A Spanish Porcine Reproductive and Respiratory Syndrome Virus 1 Strain Is Highly Virulent in Pigs Under Experimental Conditions

**DOI:** 10.1155/tbed/4847981

**Published:** 2025-05-23

**Authors:** Paula Curto, Ariadna Pey, Alba Trabal, Elisabet Aulinas, Jordi Baliellas, Gustavo Ramírez, Jéssica Molín, Larysa Muzykina, Maria Montoya, Ramona N. Pena, Lorenzo Fraile, Ana M. M. Stoian

**Affiliations:** ^1^Department of Animal Science (ETSEA), Universitat de Lleida-AGROTECNIO-CERCA Centre, Lleida, Spain; ^2^A.M. Animalia Bianya, S.L., La Vall de Bianya, Spain; ^3^Grup de Sanejament Porcí (GSP), Lleida, Spain; ^4^Molecular Biomedicine Department, The Margarita Salas Center for Biological Research (CIB) of the Spanish National Research Council (CSIC), Madrid, Spain

**Keywords:** experimental infection, high-virulence, pig, porcine reproductive and respiratory syndrome virus

## Abstract

Porcine reproductive and respiratory syndrome (PRRS) remains one of the most important infectious diseases affecting the global pig industry. The emergence of highly pathogenic PRRS virus 1 (PRRSV-1) strains, first detected in northeastern Spain in 2020, poses new challenges for effective disease control. Despite their severe impact under field conditions, a comprehensive experimental characterization of these virulent strains has been lacking. In this study, we analyzed the genetic characteristics of the Lleida 029_22 strain, collected during a severe PRRSV-1 outbreak, and evaluated its pathogenicity in pigs. Phylogenetic analysis revealed that the Lleida 029_22 strain belongs to a new clade describing multiple strains isolated during a PRRSV-1 Rosalia-related outbreak, with the highly pathogenic Italian strain PR40 identified as a common ancestor. *In vitro*, this strain replicated efficiently in porcine alveolar macrophages and PAM-KNU cells, but not in MARC-145 cells. To characterize the pathogenicity of this strain, 8-week-old piglets were inoculated with a 2 × 10^5^ TCID_50_ dose of the Lleida 029_22 strain via two inoculation routes—intramuscular (IM) and intranasal (IN). The IM-infected pigs exhibited high levels of viremia and virus shedding, with 100% mortality recorded by 14 days post-infection (dpi). These animals also showed significantly elevated levels of pro-inflammatory cytokines, particularly IL-6, and severe histopathologic lung lesions, correlating with the observed severe disease outcome. In contrast, IN-infected pigs exhibited lower mortality (30%) and moderate clinical signs, with the surviving pigs fully recovering by the end of the 63-day observation period. These pigs also exhibited prolonged viremia and viral shedding, with lower levels of pro-inflammatory cytokines and neutralizing antibodies from 28 dpi. The IN infection closely mimicked the disease outcomes observed in Spanish farms experiencing outbreaks due to the high-virulence Rosalia strain, while the IM infection highlighted the risks of iatrogenic transmission.

## 1. Introduction

Porcine reproductive and respiratory syndrome (PRRS) remains one of the most economically important diseases affecting the swine industry. This disease is caused by the PRRS virus (PRRSV), a member of the *Arteriviridae* family [1], and comprises two species: *Betaarterivirus europensis* (virus name PRRSV-1; formerly known as *Betaarterivirus suid 1*), which is predominant in Europe, and *Betaarterivirus americense* (virus name PRRSV-2; formerly known as *Betaarterivirus suid 2*) [2], which predominates in America and Asia [3]. PRRSV has an enveloped positive-sense RNA genome ~15 kb in length, encoding 11 known open reading frames (ORFs) [4]. PRRSV is characterized by its high mutation and recombination rates, leading to rapid evolution and a highly diverse genetic and antigenic viral population [5]. This variability is observed not only for PRRSV-2 [6, 7] but also for PRRSV-1 [8, 9], which has been classified into four subtypes [10, 11] based on phylogenetic analysis of ORF5 and ORF7 sequences. This high level of variability is associated with the unpredictable and variable efficacy of modified live vaccines, which are commonly used to control PRRS [12].

In 2020, a highly virulent PRRSV-1 strain, commonly named “Rosalia” [13], emerged in the northeastern part of Spain [14] and spread rapidly nationwide. However, official information regarding its dissemination and impact on the Spanish pig production sector is lacking, as the disease is not under official control. One peer-review study described the effects of this newly emerged high-virulence PRRSV-1 strain within a Spanish pig production system previously infected with “classical” PRRSV-1 strains [15]. This study observed a rapid spread of the new strain, with an impact similar to that seen in farms encountering PRRSV for the first time. The resulting impact on productivity and health was severe, taking several months for the system to return to normality [15]. Given this context, it is likely that the impact at a national level has been high. Data supporting this hypothesis consists of a 5.1% increase in mortality during the rearing period (nursery and fattening), as reported at the national level from 2020 to 2022 [16]. While this increase cannot be attributed solely to the emergence of the highly virulent PRRSV-1 Rosalia strain, the virus is likely a key driver [17]. Additionally, five million piglets for fattening were imported into Spain from other European pig-producing countries between 2021 and 2023, with no other major new pig diseases reported in Spain during this period. Sequencing of different PRRSV-1 isolates collected during a Rosalia-related outbreak in Spain suggests that this highly virulent strain may be derived from the high virulence PRRSV-1 PR40 strain described in Italy [18], after a genetic drift and several recombination events with other PRRSV-1 strains [14]. Nevertheless, a thorough epidemiological study is needed to decipher its evolution and to assess the prevalence of this highly virulent strain in populations previously affected by classical PRRSV-1 strains.

Several routes of infection have been described for PRRSV, including intranasal (IN), oral, intramuscular (IM), intrauterine, or vaginal routes [3, 19]. The virus causes a wide range of clinical presentations, from asymptomatic cases to severe forms with high mortality rates. The severity of the disease depends on factors such as the virulence of the strain, health status of the farm, herd immunity, and management practices. Traditionally, PRRSV was known for causing reproductive failure in sows, respiratory issues, and immune suppression in pigs of all ages [20]. However, the emergence of highly virulent PRRSV strains in Europe, America, and Asia has led to more severe outcomes, including high morbidity and mortality (exceeding 20%, even in adult pigs), prolonged high fever, and severe respiratory disease. High-virulence strains have also been associated with severe cyanosis of the ears and tail, conjunctival hyperemia, and diarrhea [8, 18, 20, 21]. Under field conditions, the high-virulence PRRSV-1 Rosalia strain causes high abortion rates and mortality in sows and increased mortality in weaners and growing pigs (>20%) [14, 15], but its characterization under experimental conditions remains incomplete. Such characterization is urgently needed to develop suitable models for testing the efficacy of existing modified live PRRSV vaccines, which are typically based on older strains, and to evaluate the efficacy of next-generation PRRSV vaccine candidates.

The aim of this study was to thoroughly characterize a highly virulent PRRSV-1 strain isolated during a Rosalia-related outbreak both *in vitro* and in pigs under experimental conditions using two different infection routes—IM and IN—which were selected due to their common use in the literature [6, 7, 9, 22–25]. Moreover, the IN infection model can mimic the natural route of PRRSV infection and can therefore be used in vaccine efficacy studies. Clinical signs and mortality were thoroughly recorded throughout the experimental infection, and lesions in necropsied animals were grossly, microscopically, and immunohistochemically evaluated. Moreover, viral loads in serum, saliva, nasal swabs, rectal swabs, and tissues were assessed to understand viral kinetics, and virus isolation in cell culture was carried out to determine the infectious potential of several secretions and excretions. Finally, immune responses were analyzed by measuring PRRSV specific and neutralizing antibody responses and serum cytokine levels to better understand their role in disease development.

## 2. Materials and Methods

### 2.1. Cells and Virus Strain

Primary alveolar macrophages (PAMs) collected from 5-week-old pigs by lung lavage, immortalized PAMs (PAM-KNU; Applied Biological Materials Inc.), and African green monkey kidney (MARC-145) cells were used in this study. PAMs and PAM-KNU cells were cultured in RPMI 1640 medium, while MARC-145 cells were maintained in Dulbecco's Modified Eagle Medium (DMEM). Both media were supplemented with 10% fetal bovine serum (FBS), 100 U/mL penicillin and 100 mg/mL streptomycin (Gibco). Additionally, the growth medium for PAM-KNU cells was further supplemented with 1 mM sodium pyruvate, nonessential amino acids, 10 mM HEPES, 200 µg/mL Gentamicin (Gibco) and 400 ng/mL Dexamethasone (Sigma-Aldrich). All cell cultures were maintained at 37°C in a humidified atmosphere with 5% CO_2_.

A PRRSV-1 strain, Lleida 029_22 (GenBank accession number: PQ337361), was obtained from a serum sample collected during a severe Rosalia-related outbreak in Catalonia, Spain, in December 2022. Virus isolation and propagation were performed using PAM-KNU cells (as described below), as the strain did not replicate in MARC-145 cells. To minimize *in vitro* selective pressure and preserve genetic integrity, virus stock from passage two (P2) was used in all the experiments described here. The virus stock was confirmed by PCR to be positive for PRRSV-1 and negative for other pathogens, including PRRSV-2, porcine circovirus types 2 and 3, porcine parvovirus, swine influenza virus, and *Mycoplasma suis*.

### 2.2. Whole-Genome Sequencing and Phylogenetic Analysis

The PRRSV-1 Lleida 029_22 strain was subjected to whole-genome sequencing at PathoSense BV, Ghent, Belgium, following their standard procedure. Briefly, nucleic acid was extracted from the P2 virus stock as described elsewhere [26, 27] but with the inclusion of PRRSV primers to enrich for the entire genome. Sequencing was performed on a PromethION device using R10 chemistry. Reads were base called and demultiplexed using MinKNOW software (Oxford Nanopore Technologies). The whole-genome *de novo* assembly was generated using Canu v2.2 [28], with additional polishing performed using Medaka v1.11.1 (Oxford Nanopore Technologies) and further manually curated. The assembled whole-genome sequence together with other PRRSV-1 whole-genome sequences (*n* = 28) retrieved from GenBank were used for sequence alignment and phylogenetic analysis. The multiple sequence alignment was generated using the MUSCLE algorithm in MEGA11 to evaluate sequence variation and similarity. A phylogenetic tree was constructed using the maximum likelihood method based on the general reversible time (G + I) model, and bootstrap values were calculated from 1000 replications (MEGA11).

### 2.3. Multistep Growth Curve

MARC-145, PAM-KNU, and PAMs were seeded into 24-well plates in their respective growth media. After 24 h, the cells were infected with the PRRSV-1 strain Lleida 029_22 at a multiplicity of infection (MOI) of 0.1. Following a 1 h incubation at 37°C to allow for viral adsorption, the virus inoculum was removed, the cells were washed with Dulbecco's phosphate-buffered saline (DPBS; Gibco) and fresh media was added to each well. Cell supernatants were collected at 0, 1, 6, 24, 48, and 72 h post-infection (hpi) and stored at –80°C for further analysis. In parallel, infected cells were stained following the protocol described below.

For titration of the cell culture supernatants, 10-fold serial dilutions of each sample were prepared and added to PAM-KNU cells seeded in 96-well plates. After incubation for 3 days at 37°C, the cells were stained using the protocol described below. Each sample was titrated in triplicate, and the 50% tissue culture infectious dose (TCID_50_/mL) was calculated using the Spearman and Kärber method [29, 30].

### 2.4. Immunofluorescence Assay for PRRSV Detection

PRRSV detection in cell culture was performed using an immunofluorescence antibody assay (IFA). Infected cells were fixed with 4% paraformaldehyde for 10 min at room temperature (RT), followed by permeabilization with 0.1% Triton X-100 in DPBS for 5 min at RT. After washing with DPBS, cells were blocked with 10% goat serum (Sigma-Aldrich) in DPBS for 30 min at RT. The cells were then washed again in DPBS and stained with the 1AC7 PRRSV N-protein-specific monoclonal antibody (Gold Standard Diagnostics). Following a 1 h incubation at RT, cells were washed with DPBS and incubated for 30 min at RT with Alexa Fluor 594 goat anti-mouse IgG antibody (Invitrogen) and Hoechst 33342 (Invitrogen) for nuclear visualization. After a final wash with DPBS, the cells were visualized using a fluorescence microscope (Nikon ECLIPSE Ts2R).

### 2.5. Animal Study Design

Twenty-five 8-week-old PRRSV-naïve male pigs were randomly assigned to three groups and housed in separate ABSL2 facilities. After a 1-week acclimation period, pigs in Group 1 (*n* = 10) and Group 2 (*n* = 10) were inoculated with 2 × 10^5^ TCID_50_ of the PRRSV-1 strain Lleida 029_22. Group 1 received the virus intramuscularly (2 mL, administered into the neck muscle), while Group 2 received the virus intranasally (1 mL per nostril via a mucosal atomization MAD device). Animals in Group 3 (*n* = 5) were kept as negative controls throughout the entire experimental study.

Clinical assessments were performed daily, including monitoring behavior, body condition, respiratory signs (dyspnea, cough, and nasal discharge), and digestive signs. Rectal temperatures were recorded daily for the first 10 days and continued if a fever (≥39.5°C) was observed until the animal's condition improved. Clinical observations were scored from 0 (no signs) to 3 (severe signs), while rectal temperatures were scored as follows: 0 (<39.5°C), 1 (39.5–40.5°C), 2 (40.5–41.5°C), and 3 (>41.5°C). An overall clinical score was calculated for each pig by averaging all the clinical sign scores.

Serum, saliva, nasal swabs, and rectal swabs were collected at 0, 3, 7, 14, 21, 28, 35, 42, 49, 56, and 63 days post-infection (dpi) and stored at −80°C until further testing.

Pigs were humanely euthanized at 63 dpi, or earlier if presenting with moribund conditions, via an intravenous overdose of pentobarbital sodium solution. Thorough necropsies were performed on the euthanized pigs or those found dead, and gross lung lesions were scored on a scale from 0 to 3, reflecting no lesion (0), mild interstitial pneumonia (1), moderate multifocal interstitial pneumonia (2), or severe interstitial pneumonia (3) (adapted from [31]). Fresh tissues, including lungs, tonsils, mediastinal lymph node, superficial inguinal lymph node, spleen, and liver were collected to assess viral loads. Formalin-fixed tissues, including lung, tonsil, mediastinal lymph node, superficial inguinal lymph node, and spleen, as well as brain samples from animals exhibiting neurological signs were preserved for histopathological and immunohistochemical analyses.

### 2.6. Generation of Tissue Homogenates

Tissues collected during necropsy were minced and passed through a cell strainer in the presence of DPBS supplemented with 1% penicillin/streptomycin and amphotericin B (Gibco). The resulting suspension was centrifuged at 5000 × g for 30 min at 4°C. The supernatant was carefully collected and kept on ice. The pellet was resuspended in DPBS containing antibiotics and antimycotics, then subjected to three freeze–thaw cycles. After the final freeze–thaw cycle, the suspension was centrifuged again, and the clarified supernatant was combined with the previously collected supernatant to generate a tissue homogenate. The homogenates were stored at −80°C for downstream analysis.

### 2.7. Measurement of Viral Load by RT-qPCR

Viral RNA was extracted from serum, saliva, nasal swabs, rectal swabs, and tissue homogenates using the MagMAX CORE Nucleic Acid Purification kit (Applied Biosystems) following the manufacturer's instructions. The presence of PRRSV RNA in the samples was assessed using the VetMAX PRRSV EU & NA 2.0 kit (Applied Biosystems), according to the provided protocol. All RT-qPCR reactions were carried out on a 7500 Fast Real-Time PCR system (Applied Biosystems) in a 96-well format, following the recommended cycling parameters. Each run included both negative and positive controls for consistency and validation. Samples with a threshold cycle (*C*_T_) value ≥40 were considered negative. The RT-qPCR results were expressed as 40-*C*_T_ values. Moreover, the area under the curve (AUC_p_) was calculated over the first 14 days after infection using the trapezoidal method.

### 2.8. Cytokine Detection in Serum

Serum samples collected on days 0, 3, 7, and 12–14 post-infection were analyzed for cytokine levels. Interferon *α* (IFN*α*) concentrations were measured using a porcine IFN*α* sandwich ELISA kit (Sigma-Aldrich), while interleukin-1*α* (IL-1*α*), IL-1*β*, IL-2, IL-6, and IL-12 levels were quantified using the MILLIPLEX Porcine Cytokine/Chemokine Magnetic Bead Panel (Sigma-Aldrich) on a Luminex 200 system (Merck), following the manufacturer's instructions. Standards provided in each kit were used to determine cytokine concentrations. The final concentration of each cytokine was calculated as the mean of two technical replicates and expressed in pg/mL.

### 2.9. Detection of PRRSV-Specific Antibodies

PRRSV-specific antibodies in serum samples collected until 63 dpi were detected using the PRRS X3 ELISA antibody kit (IDEXX Laboratories) in accordance with the manufacturer's instructions. Results were expressed as sample-to-positive (S/P) ratios, with samples exhibiting an S/P ratio of 0.4 or higher considered positive for PRRSV-specific antibodies.

### 2.10. Detection of PRRS Virus-Neutralizing Antibodies

PRRSV-neutralizing antibody titers in serum samples were determined using a virus neutralization assay in PAM-KNU cells. Heat-inactivated serum was serially diluted 2-fold starting from 1:4 and incubated with 400 TCID_50_ of PRRSV-1 Lleida 029_22 strain for 1 h at 37°C to allow antibody binding to the virus. Following incubation, the antibody-virus mixtures were transferred to PAM-KNU monolayers in 96-well plates and incubated for 3 days at 37°C. The cells were then fixed and stained using the IFA protocol, and the infected cells were observed under a fluorescence microscope (Nikon ECLIPSE Ts2R). The virus-neutralizing antibody titer was defined as log_2_ of the highest serum dilution that resulted in a 90% reduction in fluorescent foci compared to the control wells.

### 2.11. Virus Isolation From Animal Samples

Serum, saliva, nasal and rectal swabs, and tissue homogenate samples that tested positive by RT-qPCR were used for virus isolation. Each sample was 2-fold serially diluted starting from 1:5 and inoculated onto PAM-KNU monolayers for 2 h at 37°C. After incubation, the inoculum was removed, the cells were washed with DPBS, and fresh media was added to each well. Following a 3-day incubation, the cells were fixed and stained following the IFA protocol, and the samples showing fluorescent foci at any dilution were considered positive for virus isolation. Positive serum samples were further titrated using 10-fold serial dilutions on PAM-KNU cells to determine the quantity of infectious virus, expressed as log_10_ (TCID_50_/mL).

### 2.12. Histopathological Evaluation

Tissue samples collected during necropsy were processed using standard histopathological techniques, and slides were stained with hematoxylin and eosin. Two board-certified veterinary pathologists, who were blinded to the experimental groups, evaluated the microscopic lesions. Lung sections were scored on a 5-point scale based on the distribution and severity of interstitial pneumonia: 0 (no microscopic lesions), 1 (mild and focal interstitial pneumonia), 2 (moderate and multifocal interstitial pneumonia), 3 (moderate and diffuse interstitial pneumonia), and 4 (severe and diffuse interstitial pneumonia) [31, 32]. The severity of the lesions in the lymphoid organs was scored as follows: 0 (without lesions), 1 (light hyperplasia of lymphatic follicles, without depletion or cellular necrosis), 2 (hyperplasia of lymphatic follicles with multifocal cellular necrosis and light lymphoid depletion), 3 (moderate hyperplasia with multifocal cellular necrosis and moderate lymphoid depletion), and 4 (severe follicular hyperplasia with abundant, diffuse cellular necrosis and moderate-to-severe intensity lymphoid depletion) (adapted from [32].

### 2.13. Immunohistochemistry for PRRSV Antigen Detection

PRRSV-specific antigen was detected in tissue sections using an immunohistochemical method. Tissue sections (3 *µ*m) were mounted on adhesive glass slides (Citotest Scientific), deparaffinized, and rehydrated through a graded alcohol series. Endogenous peroxidase activity was blocked with 3% hydrogen peroxide in methanol for 30 min. The slides were then rinsed in PBS and subjected to antigen retrieval using 0.1% protease XIV (Sigma-Aldrich) for 30 min at 37°C. After washing, blocking of nonspecific binding was performed for 30 min at RT with 10% goat serum (Sigma-Aldrich) in PBS. The PRRSV N protein-specific 1AC7 monoclonal antibody (Gold Standard Diagnostics) was applied to the slides and incubated overnight at 4°C using a Sequenza Immunostaining System (Epredia). After rinsing with PBS, the slides were incubated with polymeric horseradish peroxidase (HRP)-linked goat anti-mouse/rabbit IgG (Neo Biotech) for 30 min and developed using DAB chromogen from the NeoStain Poly 1-Step with DAB kit (Neo Biotech), according to the manufacturer's instructions. The samples were then counterstained with Mayer's hematoxylin (Sigma-Aldrich), and coverslips were applied using DPX mounting medium (Sigma-Aldrich). PRRSV PCR-positive tissues were included as positive controls, while sections where the primary antibody was replaced with PBS served as negative controls.

### 2.14. Statistical Analysis

Data analysis was performed using JMP Pro, Version 17.0.0 (SAS Institute Inc., Cary, NC, USA, 1989–2019). The normality of the data was assessed with the Shapiro-Wilk test. Depending on the data distribution, pairwise comparisons between the three experimental groups at each time point were performed using either one-way ANOVA for normally distributed variables or the nonparametric Wilcoxon test for non-normally distributed variables. Moreover, the area under the curve until 14 days post-infection (AUC_p_) for serum, saliva, nasal and rectal swabs, and ELISA was compared between the three experimental groups using parametric (ANOVA test) or nonparametric test (Wilcoxon text) depending on the normality of this variable after carrying out the Shapiro-Wilk test. On the other hand, histology scores from control and IN-infected animals that died at 63 dpi were analyzed using a pairwise comparison as previously described for AUC_p_. To account for the nonindependence of observations collected at different time points for the same animals, a linear mixed model was also carried out to analyze data from the first 14 days of the study, considering group and day as fixed effects and animal as a random variable, including rectal temperatures, clinical scores, RT-qPCR results from serum, saliva, nasal and rectal swabs, and ELISA results. Statistical significance was defined as a *p*-value of less than 0.05, while a statistical tendency was described for *p*-values between 0.05 and 0.1. All figures were generated using GraphPad Prism (Version 10.1.1).

## 3. Results

### 3.1. Phylogenetic Characterization and Nsp2 Amino Acid Analysis of the PRRSV-1 Strain

**Figure 1:**
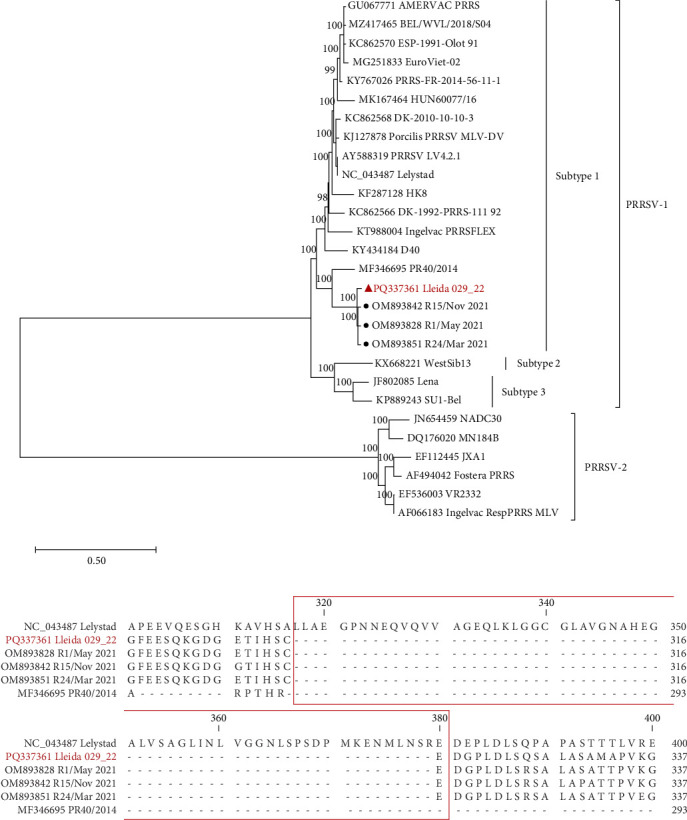
Phylogenetic analysis and Nsp2 amino acid sequence alignment of the PRRSV-1 strain Lleida 029_22. (A) Maximum likelihood phylogenetic tree constructed from whole-genome sequences using the general time reversible model and 1000 bootstrap replications. The Lleida 029_22 strain used in this study is indicated by a triangle (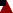) and representative strains collected during a Rosalia-related outbreak are indicated with a circle (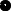). (B) Partial Nsp2 amino acid sequence alignment of Lleida 029_22, representative strains collected during a Rosalia-related outbreak, the high-virulence PR40 strain, and the prototype strain, Lelystad. Sequence alignment was performed using CLC Genomics Workbench 24.0.1. Nsp2, nonstructural protein 2; PRRSV-1, Porcine reproductive and respiratory syndrome virus 1.

The PRRSV-1 strain, Lleida 029_22 (Figure 1A), was sequenced at a depth of 286x with 100% genome coverage, revealing a total genome length of 14,858 nucleotides. Phylogenetic analysis of the whole genome placed this strain within the PRRSV-1 subtype 1 lineage (Figure 1A). Moreover, Lleida 029_22 was placed in the same clade as three other strains isolated during a Rosalia-related outbreak (Figure 1A; [14], sharing 96.61%–97.26% nucleotide homology (data not shown). Notably, all these strains, including Lleida 029_22, shared a common ancestry with the highly pathogenic PR40 strain [18].

The nonstructural protein 2 (Nsp2) amino acid sequence of the Lleida 029_22 strain was further analyzed and compared with reference strains to identify possible deletions (Figure 1B). A distinct 63-amino acid deletion was observed from positions 317–379 when compared to the prototype PRRSV-1 strain, Lelystad. Interestingly, this deletion is consistent with those found in other strains isolated during Rosalia-related outbreaks (Figure 1B), suggesting a conserved genetic feature within this group. Moreover, a larger Nsp2 deletion was identified in their common ancestral strain, PR40, indicating that this genetic signature may have emerged early in the evolutionary history of this clade (Figure 1A).

### 3.2. *In Vitro* Replication of the PRRSV-1 Lleida 029_22 Strain

**Figure 2:**
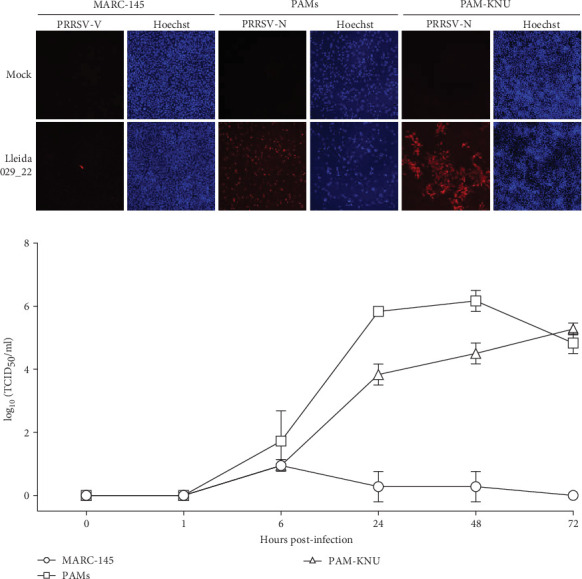
Virus growth kinetics of the PRRSV-1 Lleida 029_22 strain in MARC-145, PAMs and PAM-KNU cells. Cell cultures were infected with the Lleida 029_22 strain at an MOI of 0.1. Culture supernatants were collected at the indicated timepoints and infected cells were stained with an anti-PRRSV N protein antibody. (A) The 72 h post-infection results were examined under a fluorescence microscope using a 20× objective. (B) Virus replication at each time point was quantified by titration in PAM-KNU cells. Data are presented as mean ± SD from three independent experiments. MOI, multiplicity of infection; PAM, primary alveolar macrophage; PRRSV-1, Porcine reproductive and respiratory syndrome virus 1.

The replication characteristics of the Lleida 029_22 strain was initially assessed *in vitro*. The strain replicated efficiently in PAMs and PAM-KNU cells, but not in MARC-145 cells, as demonstrated by immunofluorescent staining of PRRSV-infected cells at 72 hpi (Figure 2A). Growth curve experiments conducted in all three cell types showed similar patterns of viral titers in PAMs and PAM-KNU, though titers were slightly lower in PAM-KNU cells (Figure 2B). By 72 hpi, both PAMs and PAM-KNU cells reached comparable viral titers of ~10^5^ TCID_50_/mL. In contrast, MARC-145 cells failed to support a productive viral infection, with viral titers decreasing to undetectable levels by 72 hpi.

### 3.3. Clinical Outcomes and Mortality Rates in Pigs After Infection With the Lleida 029_22 Strain

**Figure 3:**
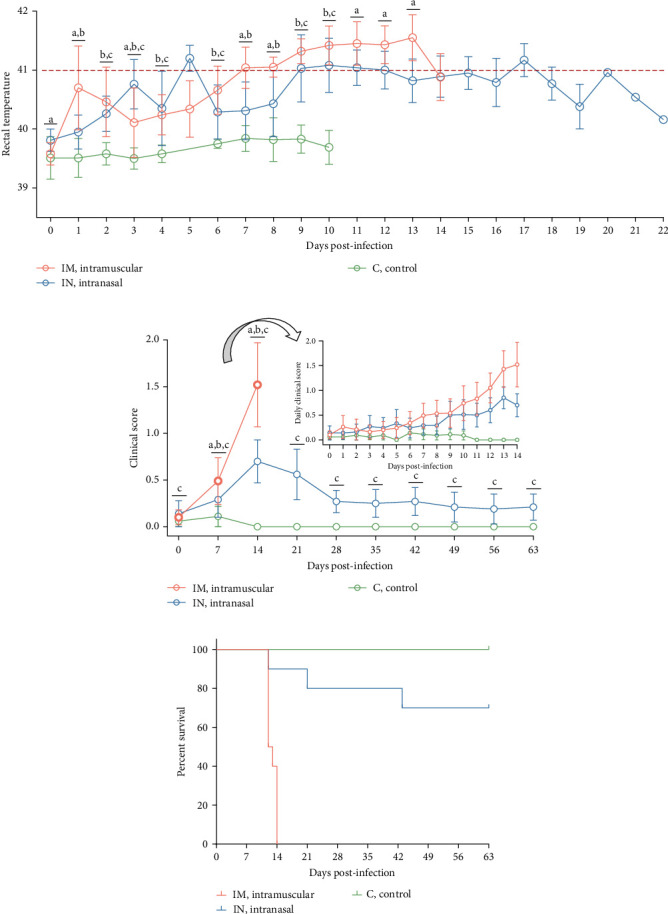
Clinical outcomes following infection with the PRRSV-1 Lleida 029_22 strain. (A) Daily rectal temperatures of pigs after infection. The red dotted line indicates the threshold for high fever (>41⁰C). Data are presented as mean ± SD. (B) Daily average clinical scores after PRRSV infection. Data are shown as mean ± SEM. Different letters indicate significant statistical differences (*p* < 0.05) between groups: IM vs IN (a), IM vs C (b) and IN vs C (c). (C) Mortality and survival curves for each experimental group. C, control; IM, intramuscular; IN, intranasal; PRRSV, porcine reproductive and respiratory syndrome; PRRSV-1, Porcine reproductive and respiratory syndrome virus 1.

The pathogenicity of the PRRSV-1 Lleida 029_22 strain was evaluated in pigs using the IM and IN infection routes. Rectal temperatures in the IM-infected pigs began to rise at 1 dpi, exceeding 41°C by 7 dpi, and remained elevated until 13 dpi. In contrast, IN-infected pigs exhibited a less pronounced febrile response, with temperatures reaching >41°C at 5 dpi, followed by intermittent fever throughout the study. The control group, however, showed no abnormal rectal temperature changes throughout the experimental period (Figure 3A).

Clinically, IM-infected pigs presented acute respiratory distress, neurological signs, severe dyspnea, and cyanosis of the ears and scrotum. IN-infected pigs primarily showed moderate and prolonged dyspnea. Additional clinical signs, including a rough hair coat, edema (notably in the limbs and neck), limb inflammation, and overall poor body condition, were observed in both infected groups but were more severe in the IM-infected animals. These severe clinical signs were predominantly observed 12–14 dpi, which coincided with the period when most pigs were either found dead or needed to be euthanized for welfare reasons. Overall, clinical scores were significantly higher in the IM-infected group at 11, 12, and 14 dpi compared to the IN-infected group (*p*  < 0.05), with the highest scores recorded at 14 dpi (Figure 3B). The IN-infected pigs exhibited lower clinical scores, peaking at 13 dpi, with a subsequent decline in clinical signs towards the end of the study, although scores remained significantly higher than those of the control group (*p*  < 0.05). No clinical signs were observed in the control group during the entire experimental period (Figure 3B).

A linear mixed model analysis, accounting for the nonindependence of these observations within the same animals across different time points, revealed statistically significant differences in rectal temperatures and clinical scores among the group and day variables during the first 14 days of the study. Notably, significant differences were observed between the IM- and IN-infected groups, as well as between each of the infected groups (IM and IN) and the control group (*p*  < 0.05).

Survival analysis revealed that all pigs in the IM-infected group succumbed to the infection by 14 dpi, resulting in a 100% mortality rate, with natural or induced deaths occurring between 12 and 14 dpi. In the IN-infected group, the mortality rate was 30% by 63 dpi, with deaths occurring at 12, 21, and 43 dpi. In contrast, all animals in the control group survived until the end of the study (Figure 3C).

### 3.4. Detection of Viremia and Viral Shedding in PRRSV-Infected Animals

**Figure 4:**
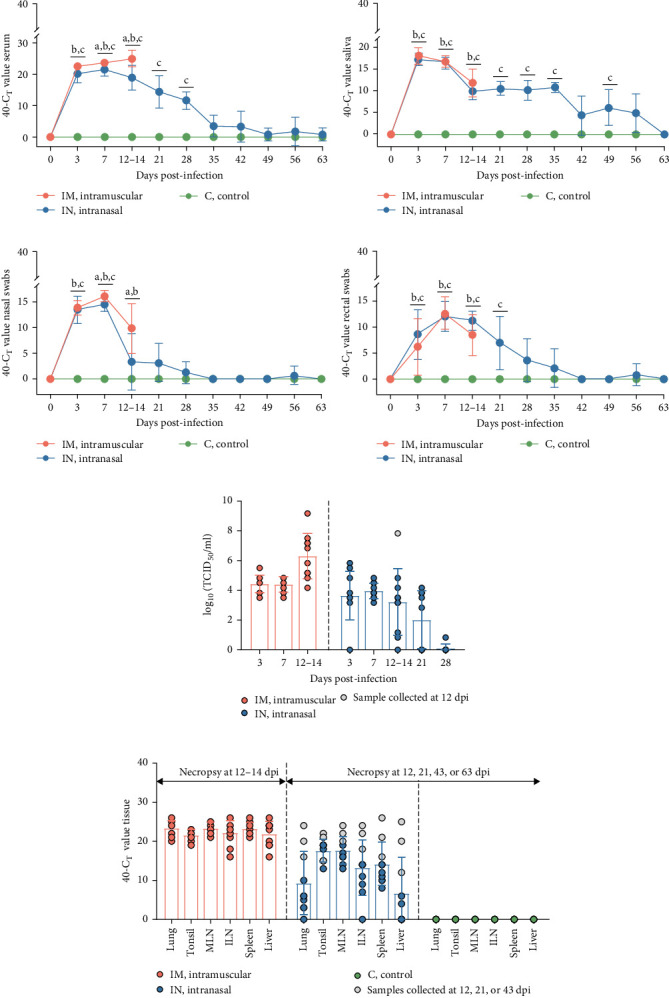
Viral load detection in serum, saliva, nasal and rectal swabs, and tissue samples. (A–D) PRRSV RNA detection in serum, saliva, nasal, and rectal swabs from pigs after PRRSV infection using RT-qPCR. Different letters indicate significant statistical differences (*p* < 0.05) between groups: IM vs IN (a), IM vs C (b) and IN vs C (c). (E) Titration of PCR and virus isolation-positive serum samples in PAM-KNU cells. (F) PRRSV RNA detection in different tissues from pigs in each experimental group using RT-qPCR. Data are presented as mean ± SD. C, control; IM, intramuscular; IN, intranasal; PAM, primary alveolar macrophage; PRRSV, porcine reproductive and respiratory syndrome.

Viremia levels following infection with PRRSV-1 Lleida 029_22 were inferred using RT-qPCR. By 3 dpi, PRRSV RNA was detectable in the serum of all infected pigs, with both IM and IN groups showing significantly higher viremia compared to the control group, which remained negative (*p*  < 0.05; Figure 4A). The IN-infected group showed a peak in viremia at 7 dpi, while the IM-infected animals had significantly higher viremia levels at both 7 and 14 dpi compared to the IN-infected and control groups (*p*  < 0.05). Thereafter, the viremia levels in the IN-infected animals gradually decreased until the end of the study but remained significantly higher than those in the control group until 28 dpi (*p*  < 0.05; Figure 4A). Additionally, the average AUC_p_ of viral load in serum was significantly higher in both the IM- and the IN-infected groups compared to the control group (*p*  < 0.05; Figure S1A).

Viral shedding was evaluated through saliva and nasal and rectal swabs by RT-qPCR. At 3 dpi, all infected animals tested positive for PRRSV in these samples. Shedding through the oral route continued in both infected groups until 14 dpi in the IM-infected group and throughout the study in the IN-infected group, with peak shedding occurring at 3 dpi (Figure 4B). Nasal shedding was observed from 3 to 14 dpi in the IM-infected group and from 3 to 28 dpi in the IN-infected animals, with a weak rebound at 56 dpi. The highest peak of nasal shedding occurred at 7 dpi for both infected groups (Figure 4C). Notably, nasal shedding was significantly higher in the IM-infected animals at 7 and 14 dpi compared to the IN-infected group (*p*  < 0.05). Viral shedding through the rectal route was detected from 3 to 14 dpi in the IM-infected group and from 3 to 35 dpi in the IN-infected group, with a weak rebound at 56 dpi. Peak rectal shedding also occurred at 7 dpi for both infected groups (Figure 4D). The average AUC_p_ of viral load in saliva, and nasal and rectal swabs was significantly higher in both infected groups compared to the control group. The average AUC of viral load in nasal swabs was also significantly higher in the IM-infected animals compared to the IN-infected ones (*p*  < 0.05; Figure S1B–D). These findings suggest that PRRSV-infected pigs began shedding the virus shortly after infection and continued to shed it over an extended period.

To account for the nonindependence of PRRS viral load measurements in either serum, saliva, nasal swabs, or rectal swabs for the same animals at different time points, a linear mixed model analysis was carried out for the first 14 days post-infection. This analysis revealed statistically significant differences across serum, saliva, and rectal swab samples during this study period. Thus, significant differences were observed between each of the infected groups (IM and IN) and the control group for saliva and rectal swabs. Furthermore, the model showed significant differences between the IM- and IN-infected groups, as well as between each infected group and the control group for serum and nasal swabs (*p*  < 0.05).

### 3.5. Detection of Infectious PRRSV in PCR-Positive Samples

**Figure 5:**
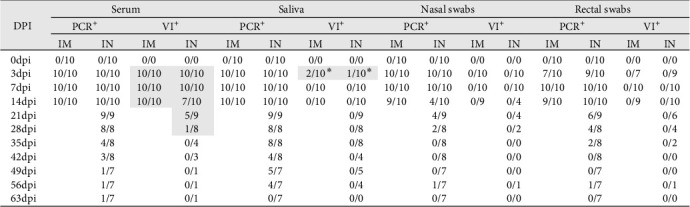
Summary of RT-qPCR and virus isolation results from different sample types collected during the experimental study. PCR results are shown as number of positive samples per total number of tested animals; VI results are shown as number of positive samples per total number of PCR-positive tested samples. *⁣*^*∗*^Positive only after two rounds of virus isolation; in grey are highlighted the positive VI results. VI, virus isolation.

To determine the presence of infectious PRRSV in serum, saliva, and nasal and rectal swabs that tested positive by RT-qPCR, a virus isolation (VI) assay was carried out in PAM-KNU cells. The results varied depending on the sample type (Figure 5). In serum samples, infectious virus was isolated from all PCR-positive samples collected from the IM-infected group between 3 and 14 dpi, showing a 100% (10/10) positivity rate. Similarly, 100% (10/10) of the serum samples from the IN-infected group tested positive between 3 and 7 dpi. Furthermore, a subset of serum samples from the IN-infected animals remained positive for PRRS infectious virus at 14 dpi (70%; 7/10), 21 dpi (55.6%; 5/9), and 28 dpi (12.5%; 1/8). Notably, infectious virus was detected in three saliva samples collected at 3 dpi from both infected groups after two passages in PAM-KNU cells. However, no infectious virus was isolated from any nasal or rectal swab samples.

The levels of infectious virus in VI-positive serum samples were quantified by titration in PAM-KNU cells and expressed as TCID_50_/mL (Figure 4E). In the IM-infected pigs, viral titers in serum samples collected between 3 and 14 dpi ranged from 10^3.5^ to 10^9.17^ TCID_50_/mL with the highest titers observed at 12–14 dpi. This peak in viral titers coincided with high mortality in the IM group by 14 dpi. In contrast, IN-infected pigs exhibited slightly lower viral titers, ranging from 10^0.83^ to 10^7.83^ TCID_50_/mL in serum collected between 3 and 28 dpi. The highest titer was observed in a pig that died at 12 dpi.

### 3.6. Cytokine Response to PRRSV-1 Lleida 029_22 Infection

**Figure 6:**
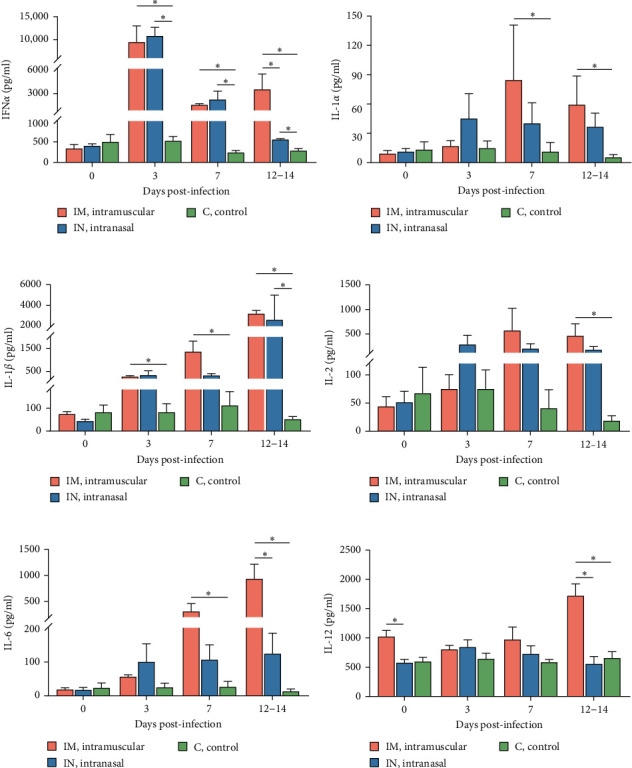
Dynamics of proinflammatory cytokines. (A–E) Levels of different cytokines were measured in serum samples over the first 14 days post-infection using either a commercial ELISA kit (IFN-*α*) or the MILLIPLEX Porcine Cytokine/Chemokine Magnetic Bead Panel (IL-1*α*, IL-1*β*, IL-2, IL-6, IL-12). Data are presented as mean ± SEM. Significant statistical difference is indicated by *⁣*^*∗*^(*p* < 0.05).

Serum cytokine levels during infection with PRRSV-1 Lleida 029_22 were measured over the first 14 days of the study, revealing significant variability among the three animal groups (Figure 6). High levels of IFN*α* were detected at 3, 7, and 14 dpi in both infected groups, with significant differences observed compared to the control group (*p*  < 0.05; Figure 6A). Notably, at 14 dpi, the IM-infected group exhibited significantly higher IFN*α* levels than the IN-infected group (*p*  < 0.05). The proinflammatory cytokines IL-1*α*, IL-12, and IL-6 followed similar patterns (Figure 6B, D, and E). In general terms, in the IN-infected group, cytokine levels increased at 3 dpi and remained stable at 7 and 14 dpi. In contrast, the IM-infected group showed increased cytokine levels at 7 and 14 dpi, which were significantly higher than those in the control group (*p*  < 0.05). At 14 dpi, IL-6 levels in the IM-infected pigs were also significantly higher compared to IN-infected ones (*p*  < 0.05). Both infected groups showed increased IL-1*β* levels post-infection (Figure 6C). IM-infected group had significantly higher IL-1*β* levels than the control pigs at 3, 7, and 14 dpi, while the IN-infected pigs showed significantly higher levels compared to the controls only at 14 dpi (*p*  < 0.05). IL-12 levels remained relatively stable across time points, with significantly higher levels detected in IM-infected pigs compared to IN-infected pigs and controls at 14 dpi (Figure 6F). Overall, the IM-infected group exhibited higher levels of proinflammatory cytokines, particularly IL-6, at 7 and 14 dpi, correlating with the severe clinical outcomes and 100% mortality observed by 14 dpi.

To account for the potential nonindependence of cytokine levels in serum collected from the same animals at different time points, a linear mixed model analysis was conducted during the first 14 days post-infection. Statistically significant differences were identified for IL-1*β*, IL-6, and IL-12 across both group and day variables, whereas a statistical tendency (*p*=0.07) was observed for IFNa for the group variable over the 14-day period. The model also identified significant differences between the IM- and IN-infected groups for the IL-12 levels, as well as between the IM-infected group and the control group for IL-1*β* and IL-12 levels (*p*  < 0.05).

### 3.7. PRRSV-Specific and Neutralizing Antibody Responses in PRRSV-1 Lleida 029_22 Infected Animals

**Figure 7:**
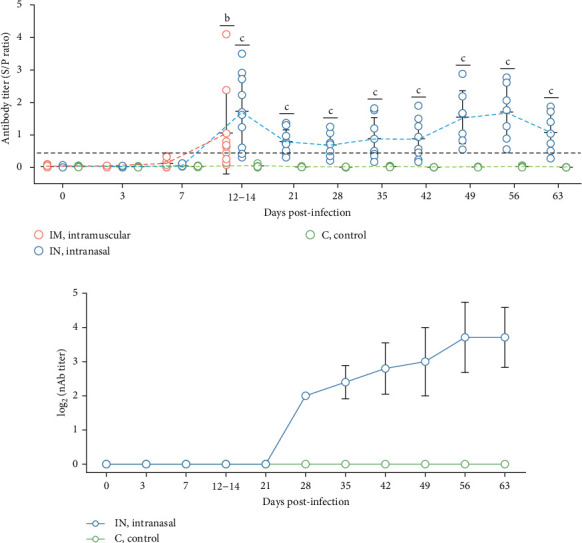
Antibody responses following infection with the PRRSV-1 Lleida 029_22 strain. (A) PRRSV-specific antibody levels in serum were measured by ELISA. A seroconversion threshold was set at an S/P ratio of 0.4, indicated by the dotted line. Different letters indicate significant statistical differences (*p* < 0.05) between groups: IM vs C (b) and IN vs C (c). (B) Neutralizing antibody (nAb) titers were assessed in PAM-KNU cells using serum samples from the IN-infected and C groups. Data are presented as mean ± SD. C, control; IM, intramuscular; IN, intranasal; PAM, primary alveolar macrophage; PRRSV, porcine reproductive and respiratory syndrome.

The presence of antibodies against PRRSV was evaluated using a commercial ELISA designed to detect antibodies against both PRRSV-1 and PRRSV-2 strains (Figure 7A). There was considerable variability in antibody levels among both infected groups. By 14 dpi, 70% of the IM-infected pigs and 90% of the IN-infected pigs had seroconverted, showing significantly higher S/P ratios compared to the control group (*p*  < 0.05). At 21 dpi, antibody levels in IN-infected animals began to decline but remained relatively stable until 42 dpi. Subsequently, levels increased until 56 dpi, followed by a slight decline towards the end of the study (63 dpi). The control group remained serologically negative (S/P <0.4) throughout the animal experiment. The AUC_p_ analysis of PRRSV-specific antibody levels over the first 14 days revealed significant differences between the infected groups and the control (*p*  < 0.05), although there was no significant difference between the two infected groups (Figure S1E). To account for potential nonindependence of PRRSV ELISA antibody levels from the same animals across time points, a linear mixed model was applied during the first 14 days post-infection. This analysis identified statistically significant differences across groups and time points within the first 14 days, particularly between the IN-infected and control groups (*p*  < 0.05).

Neutralizing antibody levels against the homologous PRRSV strain were also measured in serum from IN-infected animals and controls (Figure 7B). In the IN-infected group, neutralizing antibody titers as low as 2 log_2_ were detected starting at 28 dpi. These titers gradually increased, peaking at 56 dpi with an average value of 3.71 log_2_. There was, however, some variability in neutralizing antibody titers among pigs at the same timepoint. The appearance of neutralizing antibodies coincided with the clearance of infectious virus in serum after 28 dpi (Figure 4B). No neutralizing antibodies were detected in the control animals.

### 3.8. Detection and Isolation of PRRSV-1 Lleida 029_22 From Tissue Homogenates

Viral loads in different tissues from pigs infected with PRRSV-1 Lleida 029_22 strain were determined using RT-qPCR (Figure 4F). In the IM-infected group, viral loads were relatively consistent across all tissues, with the highest loads observed in the lungs, mediastinal lymph node, and spleen. In contrast, the IN-infected group exhibited variability in tissue viral loads, with the highest levels detected in the tonsil and mediastinal lymph node. As expected, the overall viral load in the IN-infected animals was lower than in the IM-infected ones, likely due to the different post-infection time points when necropsies were performed. The high variability in viral loads among tissues from IN-infected pigs may also be linked to the different times of death (12, 21, and 43 dpi, represented in gray, and 63 dpi: Figure 4F). No viral RNA was detected in any of the samples collected from the negative control pigs.

Virus isolation from PCR-positive tissues was attempted using PAM-KNU cells (Table 1). The success rate of virus isolation varied among tissues and between the two infected groups. In the IM-infected group, virus isolation was successful in 100% of the lung (10/10) and inguinal lymph node (10/10) samples and in 90% of the spleen (9/10) and liver (9/10) samples. Lower success rates were observed in the tonsil and mediastinal lymph node samples, with virus isolated from only 40% (4/10) and 50% (5/10) of these tissues, respectively. In the IN-infected group, successful virus isolation was achieved only in tissue homogenates from some animals that died earlier in the study (12, 21, or 43 dpi), regardless of tissue type.

### 3.9. Pathological and Immunohistochemical Analysis of PRRSV-Infected Tissues

**Figure 8:**
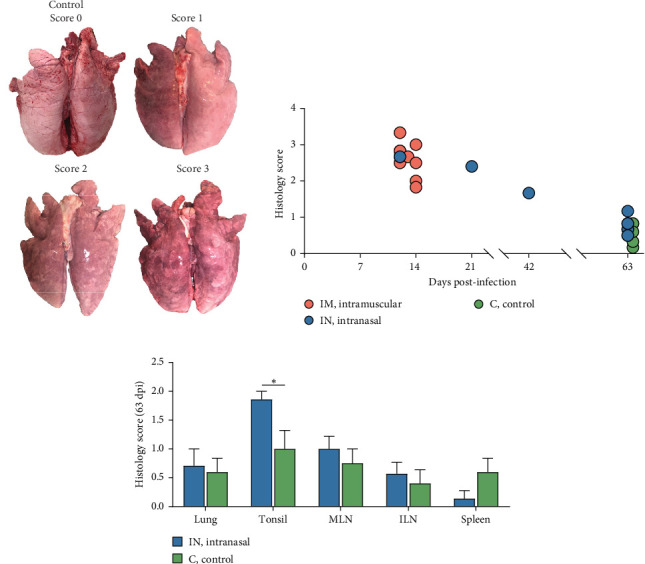
Gross and histopathological evaluation of tissues following infection with the highly virulent PRRSV-1 Lleida 029_22 strain. (A) Representative images of lung tissues showing healthy and progressively severe pulmonary lesions. (B) Histology scores for each pig, correlated with their time of death. (C) Histology scores by tissue type for all IN-infected pigs and negative controls that survived until 63 dpi. Data are presented as mean ± SD. IN, intranasal; PRRSV-1, Porcine reproductive and respiratory syndrome virus 1.

Several macroscopic lesions were observed during necropsy of the IM- and the IN-infected animals that died between 12 and 21 dpi. These lesions included generalized cyanosis, limb and generalized subcutaneous edema, moderate to severe generalized lymphadenomegaly, splenomegaly with or without follicular hyperplasia, and hemorrhagic lesions in the stomach. Gross lung lesions included partial failure to collapse, multifocal parenchymal consolidation, mottled areas of red-brownish discoloration, and occasional areas of atelectasis, all consistent with varying severity of PRRSV-associated interstitial pneumonia (Figure 8A). Overall, more severe lung lesions were observed in the IM- and IN-infected animals that died before 43 dpi. Moreover, 20% of the intranasally PRRSV-infected animals presented lesions compatible with a secondary bacterial infection, such as abscesses in the submandibular, mandibular, and mediastinal areas, chronic fibrinous diffuse pericarditis, and chronic purulent polyarthritis. No macroscopic lesions were observed in the animals from the negative control group.

Histopathological analysis was performed on lungs, tonsils, mediastinal and inguinal lymph nodes, and spleen. Lesion severity was scored on a scale from 0 to 4, with scores averaged based on the time of death (Figure 8B). Overall, lesions were more severe in animals that died in the early stages of infection (12–14 and 21 dpi), with histopathology scores ranging from 1.83 to 3.33 (Figure 8B). In contrast, animals euthanized at 63 dpi exhibited lower lesion scores, ranging from 0.17 to 0.83 in the control group and from 0.50 to 1.17 in the IN-infected group. Statistical analysis revealed that the IN-infected group euthanized at 63 dpi had significantly higher histology scores in tonsil tissue compared to the control group (*p*  < 0.05), though no significant differences were observed in other tissues (Figure 8C).

**Figure 9:**
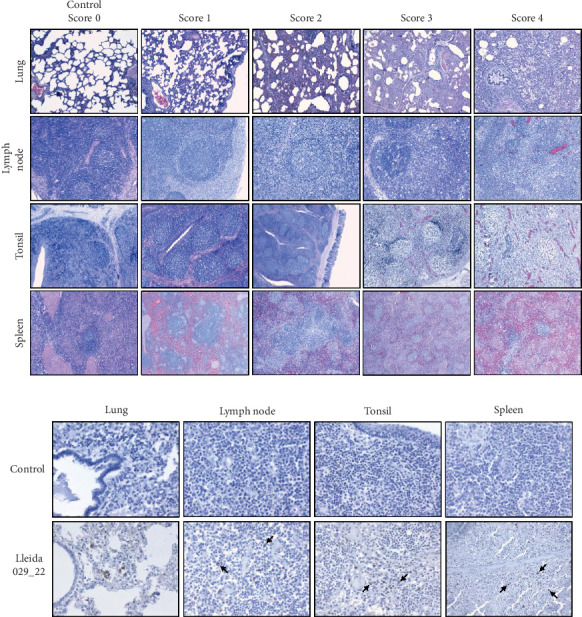
Microscopic evaluation of tissue lesions following infection with the highly virulent PRRSV-1 Lleida 029_22 strain. (A) Representative images of histopathological examination of lung, lymph node, tonsil, and spleen from negative control and infected pigs, stained with hematoxylin and eosin (10× magnification). (B) Representative images of lung, lymph node, tonsil, and spleen sections from negative control and infected animals, stained with a PRRSV N protein antibody by IHC (40× magnification). Arrows indicate positive signals. PRRSV, porcine reproductive and respiratory syndrome; PRRSV-1, Porcine reproductive and respiratory syndrome virus 1.

Microscopic evaluation of lung tissue revealed interstitial pneumonia in both infected groups, characterized by alveolar septal thickening due to pneumocyte hypertrophy and hyperplasia, along with mononuclear infiltration (Figure 9A). Fibrin, plasma, macrophage aggregates, and necrotic material were present in the alveoli, and signs of vascular damage were evident. One IM-infected animal displayed bronchopneumonia, compatible with a secondary bacterial infection. Examination of lymphoid tissues revealed significant lymphoid depletion, lymphocyte loss, and necrosis. In the lymph nodes, and to a lesser extent in the spleen, this depletion was accompanied by fibrin and erythrocyte aggregates, microthrombi, vasculitis, and vascular necrosis (Figure 9A). Brain samples from cerebral hemispheres showed multifocal mononuclear perivascular infiltrates, which were more prominent in the white matter than in the gray matter or within leptomeninges, occasionally accompanied by vasculitis. Other changes included diffuse white matter interstitial edema and gliosis, vascular congestion with endothelial prominence, occasional perivascular microhemorrhages, and microglial activation (data not shown).

Immunohistochemistry (IHC) staining was used to detect PRRSV antigens in various tissues. Intracytoplasmic positive PRRSV signals were observed in alveolar macrophages from lungs, lymph nodes, tonsils, and spleen (Figure 9B). Occasional PRRSV antigens were detected in type II pneumocytes. In the brain, positive IHC macrophages were present within the perivascular cuffs (data not shown).

## 4. Discussion

In this study, a PRRSV-1 strain, Lleida 029_22, was isolated from a nursery farm experiencing a severe Rosalia-related outbreak in Lleida, Spain. During the outbreak, the mortality rate exceeded 20%–40%, significantly higher than the average mortality rate of 8.6% observed in Spanish nurseries in 2022 [16]. Since the first description of PRRSV-1 in the 1990s, different terms have been used to describe strains that cause more severe clinical manifestations and high mortality, with “highly virulent strains” being one of the most used in the literature. This terminology is appropriate for the Lleida 029_22 strain, as “highly virulent” PRRSV strains are known to cause increased mortality during the rearing period, severe reproductive disorders, and severe interstitial pneumonia accompanied by a strong inflammatory response [20]. The main purpose of this study was to fully characterize the PRRSV-1 Lleida 029_22 strain both *in vitro* and *in vivo* and to evaluate its impact on pigs using two infection routes under experimental conditions. This characterization involved assessing its phylogenetic relations, clinical signs, virological parameters, gross and microscopic lesions, and the immune response of the animals by measuring the dynamics of proinflammatory cytokine and antibody responses.

PRRSV is characterized by its restricted cell tropism for the monocytic lineage, although this virus can also replicate in dendritic cells [4]. PRRSV strains are typically isolated and/or cultured on PAMs and MARC-145 cells *in vitro*. Highly pathogenic strains can be successfully isolated in PAMs, whereas adaptation to MARC-145 cells can be challenging [3, 33]. Given the limited availability and variability of PAMs between batches [34], there is a need to explore alternative immortalized cell lines that may be permissive to these high-virulence PRRSV strains. Therefore, the PAM-KNU cell line, an immortalized cell line susceptible to some PRRSV field strains, was used to isolate the virus and characterize it *in vitro* [35]. The PAM-KNU cell line proved permissive to PRRSV Lleida 029_22 and showed a viral growth curve similar to that observed in PAMs. Future work should focus on identifying suitable cell lines for isolating different PRRSV strains, as no single cell line has been identified yet to be universally permissive to PRRSV strains [34].

Several factors influence the capability of a virus to infect the host and the severity of the infection, including the ability of a virus to enter target cells, its replication rate, the damage it causes to host cells, and the induction of cell death or specific immune responses [36]. Phylogenetic analysis placed the PRRSV-1 Lleida 029_22 strain in a separate clade composed of strains isolated during Rosalia-related outbreaks. This newly described clade is characterized by highly virulent strains that emerged in 2020 in Spain [14, 15]. Notably, the Lleida 029_22 strain features a 63-amino acid deletion in the Nsp2-coding region (ORF1a), a deletion observed in other PRRSV-1 and PRRSV-2 virulent strains and considered a genetic marker of virulence [18, 37, 38]. However, this finding must be interpreted with caution as deletions in the Nsp2 region have also been observed in nonvirulent PRRSV strains [39]. Thus, further studies are needed to confirm the genetic differences between highly virulent and less virulent PRRSV strains, with whole genome sequencing as an essential tool for this investigation [40].

In the literature, different infection routes—IN, IM or a combination of both—have been used to develop models of PRRSV infection in pigs and pregnant sows [6, 7, 9, 22–25]. While all these routes are effective for infecting animals, significant variability in clinical outcomes has been observed under experimental versus field conditions. Thus, reproducing the high mortality rates associated with virulent PRRSV strains in experimental settings can be challenging. Some studies have successfully replicated these outcomes using virulent PRRSV-1 and PRRSV-2 strains [25], while others have not [18], likely due to differences in conditions such as the presence or absence of coinfections (bacterial and/or viral).

In the current study, the IN route was used to mimic natural infection and the IM route to simulate potential iatrogenic transmission during the administration of drugs for treating bacterial coinfections or vaccines. However, it is important to note that this extrapolation of the results seen in the IM-infected group to the possible iatrogenic transmission at the farm level should be interpreted with caution as the viral loads observed at the farm level due to iatrogenic transmission could be different than those observed in our experimental study following a 2 × 10^5^ TCID_50_ inoculation dose. Another aspect worth mentioning is the use of male pigs in our experimental infection. This was done to minimize any potential variability in terms of PRRSV outcome that can be gender-attributed, although, to the best of our knowledge, this variability has not been described in PRRSV infections and should not impact the observed results.

Clinical signs after PRRSV infection can vary widely from asymptomatic to severe disease [8, 37]. In the experimental infection described herein, both infected groups presented severe clinical signs, which were more prominent in animals infected via the IM route. These animals showed acute respiratory distress, neurological symptoms, severe dyspnea, high fever, and cyanotic ears and reached a 100% mortality rate by 14 dpi. Conversely, the IN-infected group primarily exhibited prolonged mild to moderate dyspnea, fever, and 30% mortality by 63 dpi. This clinical outcome is consistent with other studies infecting animals with high-virulence strains, which typically result in increased mortality, severe respiratory distress, and prolonged and high fever [7, 8, 18, 21].

Data from field outbreaks suggest that a mortality rate of 20% or higher can be expected in pigs of different ages when a virulent PRRSV strain is present on a farm [20], which is consistent with the results observed in the IN-infected group described in this study that received a dose of 2 × 10^5^ TCID_50_ of the PRRSV-1 Lleida 029_22 strain. It is important to note that under field conditions, higher mortality rates can be observed due to coinfections with other viruses or bacteria. However, the 30% mortality observed in the IN group can be attributed mostly to PRRSV-1 Lleida 029_22 infection since less than 20% of the intranasally infected animals showed gross lesions compatible with a secondary bacterial infection. On the other hand, the increased mortality observed with the IM-infected group, which also received a dose of 2 × 10^5^ TCID_50_ of PRRSV-1 Lleida 029_22 was unexpected and, to the best of our knowledge, not previously described in the literature [6, 22, 24]. This finding agrees with observations from swine practitioners who have reported high mortality levels in farms affected by high-virulence PRRSV-1 strains when IM administration of drugs was not conducted under strict standard operating procedures (personal communication of swine practitioners-Grup de Sanejament Porcí, Lleida, Spain). This is a call for awareness that should be taken to animal management on farms. Finally, the clinical outcomes observed in the IN infection model from this study closely mirror the situation in affected farms [15]. The PRRSV complications present in these farms may be attributed to the recent importation of approximately five million piglets for fattening into Spain from other European pig-producing countries between 2021 and 2023. This importation was partly driven by the need to mitigate the devastating effects of highly virulent PRRSV strains, particularly in sows and nursery piglets [15]. The necessity to import piglets is somewhat surprising, given that Spain has the highest sow census in Europe, with ~2.6 million sows [41]. In relation to virological parameters, a prolonged viremia was observed in the IN-infected pigs, lasting up to 63 days, as determined by RT-qPCR. This duration is notably longer than the 42–49 days of viremia usually observed with low-virulence PRRSV-1 strains [24, 42]. It is important to note that while PCR detection indicates the presence of viral RNA, it does not confirm whether this virus is infectious. In our study, the infectious PRRSV (as determined by VI in serum samples) was detected from 3 to 28 dpi in the IN-infected pigs and throughout the entire observation period (14 days) in the IM-infected animals. The highest PRRSV titers detected in serum were 10^7.83^ TCID_50_/mL in the IN-infected animals and 10^9.17^ TCID_50_/mL in the IM-infected pigs, highlighting the differences in the levels of infectious virus circulating among animals based on their inoculation route. In addition, previous studies with PRRSV strains of lower virulence reported similar infectious periods (from 3 to 28 dpi), but with lower viral loads of ~10^4.9–6.1^ TCID_50_/mL [8, 23], highlighting that highly virulent strains exhibit higher viral loads.

Viral shedding was detected in saliva, nasal swabs, and rectal swabs by RT-qPCR. Saliva showed a similar pattern to serum with prolonged PRRSV detection (63 days) and high viral loads, suggesting that saliva could be a useful alternative for monitoring viral presence in animals after infection, potentially reducing the need for blood sampling. This result is noteworthy given the growing use of oral fluids in pig disease surveillance [43]. However, infectious virus was isolated from only three saliva samples despite high viral RNA copy numbers in most samples, indicating that saliva may contain factors that hinder virus isolation [34, 44]. Finally, PRRSV was detected by RT-qPCR in nasal swabs from 3 to 28 dpi and in rectal swabs from 3 to 35 dpi, with a rebound at 56 dpi in both sample types. This suggests that nasal and rectal swabs are less reliable than blood for monitoring the presence of PRRSV-1 in infected animals. While studies on low-virulence PRRSV strains have reported a similar pattern of nasal shedding with early peaks after infection and persistence until 21–28 dpi [23, 45], research on high-virulence strains has shown high levels of nasal and rectal shedding between 14 and 21 dpi [6, 46]. However, it is important to note that these studies had shorter durations compared to the study described here. Despite this, no infectious virus was isolated from nasal and rectal swabs, which does not mean that these animals are unable to infect naïve animals. Further transmission studies are necessary to assess the infectious status of these animals and the role of different excretion routes. Another relevant point to discuss is the presence of virus in naturally dead animals due to the potential risk of dissemination of the virus during the management of carcasses. In the present study, the virus was present in most of the tissues sampled from both infected groups (IN and IM) by RT-qPCR. This finding aligns with similar tissue tropism observed in previous studies with highly virulent PRRSV strains [23, 46]. Thus, strict protocols must be followed to manage biological risks associated with handling carcasses of dead animals after PRRSV infection to prevent virus dissemination [47].

Regarding cytokine production, the pattern of IFN*α* in serum after infecting pigs with PRRSV-1 Lleida 029_22 closely mirrors that observed in other high-virulence PRRSV strains, where the IFN*α* levels increase in parallel with PRRSV viral load during the first 7 dpi [9, 48, 49]. Moreover, IFN*α* levels were significantly higher in the IM-infected group compared to the IN-infected one at 14 dpi, at which point the pigs had to be euthanized for welfare reasons. Thus, this elevated IFN*α* does not appear to influence viral clearance, suggesting that IFN*α* might be ineffective in controlling virulent PRRSV replication and that the role of this cytokine in PRRSV pathogenesis may need to be reconsidered. It must be highlighted that low-virulence PRRSV strains are known for their ability to inhibit or delay type I IFN production, including IFN*α* [9, 49–51]. Similar findings have been observed with some virulent PRRSV-1 and PRRSV-2 strains using *in vitro* systems [52–54], highlighting the contradictory information surrounding this cytokine. A critical consideration is whether studies were conducted under *in vitro* or *in vivo* conditions when comparing results. Furthermore, the results of the present experiment are in accordance with previous hypotheses [51, 48] suggesting that early elevated levels of IFN*α* may contribute to hyperthermia and aggravate the inflammatory response.

Levels of IL-1*α*, IL-1*β*, IL-2, IL-6, and IL-12 increased in both infected groups compared to the control group, but only the IM-infected group exhibited significantly higher levels of proinflammatory cytokines, particularly IL-6, IL-1*α*, and IL-1*β* at 7 and 14 dpi, and IL-2 and IL-12 at 14 dpi. These results are consistent with published literature, as virulent PRRSV strains are known to trigger a characteristic inflammatory cytokine cascade, primarily mediated by IL-1*α*, IL-1*β*, and IL-6, which is associated with evident clinical signs, fever, and severe tissue damage, especially in the lungs [20]. Interestingly, no significant changes were observed in TNF-*α* levels, unlike other reports [9, 48, 49, 55]. The observed cytokine profiles help explain the differences in clinical outcomes between the two infected groups. Previous studies support this, showing that high-virulence PRRSV strains produce earlier and higher levels of proinflammatory cytokines, which can aggravate inflammation and tissue damage compared to low-virulence strains [48, 51, 55]. Further studies will be carried out to better understand the role of both pro- and anti-inflammatory cytokines in the response to this highly virulent PRRSV strain in pigs.

PRRSV-specific antibodies appeared at 14 dpi in both infection routes, with a subsequent decline followed by a rebound peak at 56 dpi, resulting in a U-shaped antibody response profile. In previous research studies with both low- and high-virulence PRRSV strains, the appearance of antibodies was observed between 7 and 14 dpi, followed by a steady increase to a plateau, with no significant differences between low- and high-virulence strains [7, 9, 45, 48, 56]. Additionally, with other PRRSV strains, antibodies have been observed as early as 7 dpi [23, 37, 46]. However, in this study, the late induction of neutralizing antibodies (28 dpi) aligns with other studies involving different PRRSV strains [23, 57], but additional experiments are required to determine whether these levels of neutralizing antibodies are sufficient to protect against re-infection. Moreover, the appearance of neutralizing antibodies could not be associated with clinical outcomes or virological parameters during this observation period, suggesting that the role of these antibodies in viral clearance remains unclear. The outcome of PRRSV infection likely depends on factors beyond the presence of neutralizing antibodies. Nevertheless, passive transfer experiments have shown that neutralizing antibodies can confer complete protection against PRRSV [58], indicating that antigens targeted by these responses could serve as promising vaccine candidates against both PRRSV-1 and PRRSV-2 [59, 60].

The lesions observed in pigs that were found dead or euthanized for welfare reasons were consistent with PRRSV infection. Macroscopic lung lesions revealed moderate to severe interstitial pneumonia in both infected groups, with more severe manifestations in animals that succumbed during the early stages of infection (before 14 dpi). Notably, most of the IN-infected pigs did not present gross lung lesions by the end of the study, suggesting no long-term negative impact on productive performance following recovery. Previous studies have shown that interstitial pneumonia and lymph node lesions tend to resolve before the end of the experiments [7, 48, 56], supporting the idea that lesion resolution could serve as a suitable endpoint in PRRSV infection studies. As previously mentioned, interstitial pneumonia is the hallmark lung lesion caused by PRRSV [18, 45, 49], while a recent study also described suppurative bronchopneumonia as a frequent finding in field cases of high-virulence PRRSV-1 strains belonging to the same clade as the Lleida 029_22 strain [13]. However, in the current study, one animal presented microscopically bronchopneumonia and two animals presented gross lesions, likely due to a secondary bacterial infection, suggesting that while PRRSV can cause severe disease by itself, other pathogens may exacerbate its effects. Finally, animals with neurological signs presented with lymphohistiocytic meningoencephalitis with occasional vasculitis, similar to that reported in previous experimental infections with highly virulent PRRSV strains [61], though there was insufficient evidence to definitively link these findings to PRRSV-induced vasculitis in the brain. While some studies have demonstrated the presence of the virus in brain lesions using IHC with highly virulent PRRSV-2 strains [62, 63, 7], further research is needed to characterize the lesions in the central nervous system associated with high-virulence PRRSV-1 strains.

In summary, a suitable infection model was developed using a highly virulent PRRSV-1 strain (collected during a Rosalia-related outbreak) using the IN route in pigs, which closely mimics the clinical outcomes observed under field conditions (increased mortality rates, severe clinical signs, high and prolonged fever, and viremia). However, significant differences in clinical outcomes were observed based on the infection route. IM-infected pigs showed acute clinical cases with high mortality rates (100%), while IN-infected animals showed lower mortality (30%) despite severe clinical signs, with full recovery by the end of the 63-day observation period. These differences between infection routes highlight the importance of proper farm management practices, particularly in recognizing iatrogenic transmission as a potential risk that can increase the severity of outbreaks.

## Figures and Tables

**Table 1:** Summary of RT-qPCR and virus isolation results from tissue samples collected during the experimental study.

Tissue	PCR^+^		VI^+^	
	IM	IN	IM	IN
Lung	10/10	9/10	10/10	2/9^a^
Tonsil	10/10	10/10	4/10	0/10
MLN	10/10	10/10	5/10	1/10^a^
ILN	10/10	9/10	10/10	1/9^a^
Spleen	10/10	10/10	9/10	2/10^a^
Liver	10/10	5/10	9/10	1/5^a^

*Note:* PCR results are shown as number of positive samples per total number of tested animals; VI results are shown as number of positive samples per total number of PCR-positive tested samples.

^a^VI positive results on tissues collected from animals that died at either 12-, 21-, or 43 dpi.

## Data Availability

The data that support the findings of this study are available from the corresponding author upon reasonable request.

## Supporting Information

Supporting Information Figure S1. Viremia, viral shedding, and antibody levels shown as AUC_p_ over the first 14 days of the study.
