# Supplementary material for: A Spanish Porcine Reproductive and Respiratory Syndrome Virus 1 Strain Is Highly Virulent in Pigs Under Experimental Conditions
Source: Transbound Emerg Dis. 2025 May 23;2025:4847981. doi: 10.1155/tbed/4847981 (PMC12124928; doi:10.1155/tbed/4847981)
Supplement: Supporting Information — Figure S1. Viremia, viral shedding, and antibody levels shown as AUCp over the first 14 days of the study. [file 4847981.f1.pptx]

## Slide 1
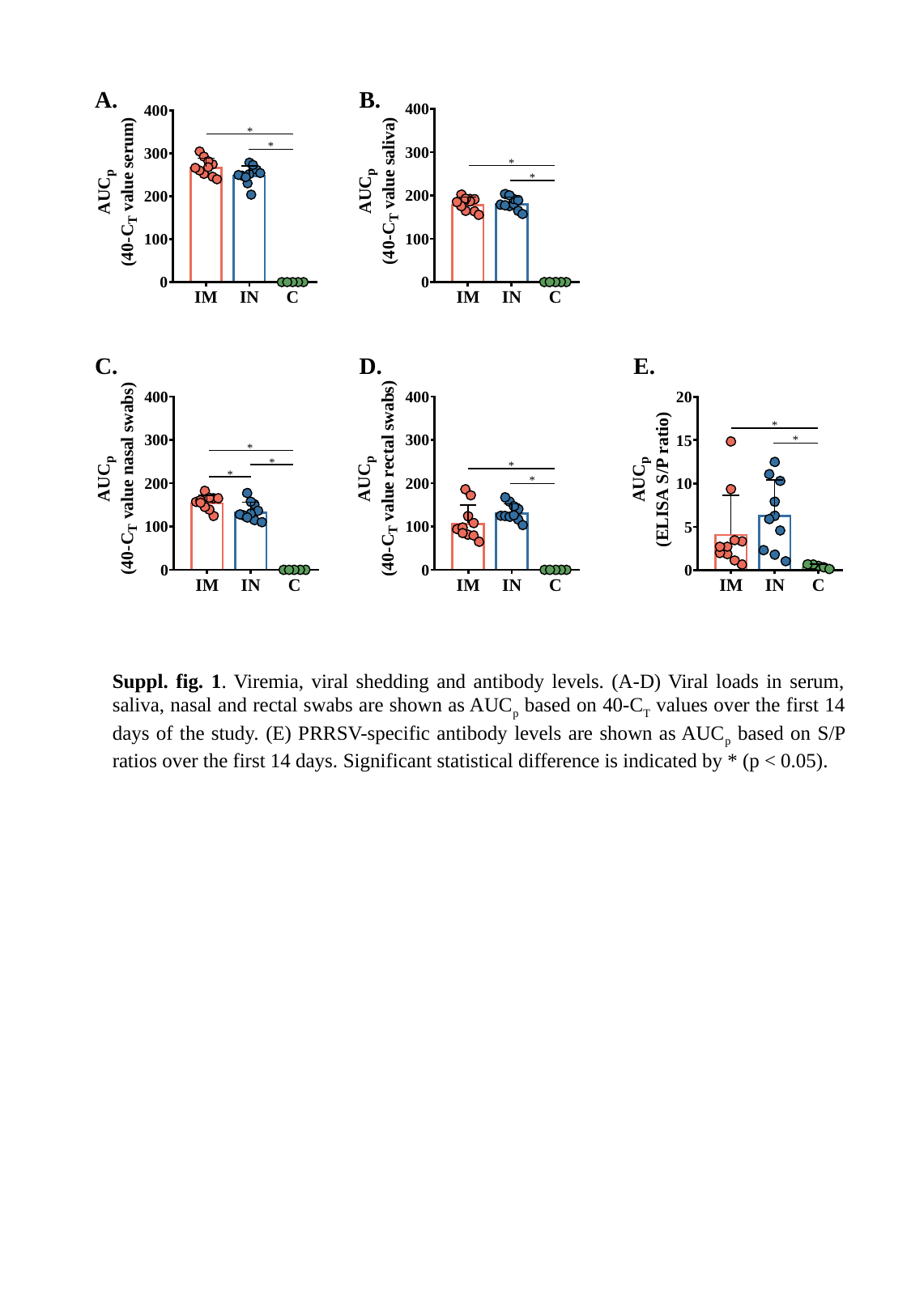

A.
B.
*
*
*
*
D.
C.
E.
*
*
*
*
*
*
*
Suppl. fig. 1. Viremia, viral shedding and antibody levels. (A-D) Viral loads in serum, saliva, nasal and rectal swabs are shown as AUCp based on 40-CT values over the first 14 days of the study. (E) PRRSV-specific antibody levels are shown as AUCp based on S/P ratios over the first 14 days. Significant statistical difference is indicated by * (p < 0.05).
